# Supplementary material for: Content-rich biological network constructed by mining PubMed abstracts
Source: BMC Bioinformatics. 2004 Oct 8;5:147. doi: 10.1186/1471-2105-5-147 (PMC528731; doi:10.1186/1471-2105-5-147)
Supplement: Additional File 2 — The original results of the above study (non-essential files are deleted to keep the file size under the limit set by BMC bioinformatics). [file 1471-2105-5-147-S2.bz2 › chilibotAdditionalFile2/dip40/712ID9732274E18049/html/PNP_RNE.html]

 


 **PNP** and **RNE** 
  
Found 60 abstracts in PubMed, retrieved 40.  
 

 What does Google say? 
 PDF only 
| .edu only 

---

**Interactive relationship** (e.g. stimulation, inhibition, etc)

**Inhibitory relationship**- We have demonstrated that the wild type RNase E  [ **RNE** ]  protein bound to polynucleotide phosphorylase  [ **PNP** ]  PNPase  [ **PNP** ]  but a truncated RNase E  [ **RNE** ]  polypeptide lacking the C terminal half did not.  Ref: 8682798 J Bacteriol, 1996
- We show that purified CspE impedes poly A mediated 3 to 5 exonucleolytic decay by PNPase  [ **PNP** ]  by interfering with its digestion through the poly A tail and also inhibits both internal cleavage and poly A tail removal by RNase E  [ **RNE** ] .  Ref: 11390393 J Biol Chem, 2001
- We demonstrate that ribosomes inhibit the RNase E  [ **RNE** ]  cleavage, 10 nucleotides downstream of the rpsO coding sequence, responsible for triggering the exonucleolytic decay of the message mediated by polynucleotide phosphorylase  [ **PNP** ] .  Ref: 9707438 EMBO J, 1998
- RNase E  [ **RNE** ]  initiates the decay of Escherichia coli RNAs by cutting them internally near their 5 end and is a component of the RNA degradosome complex, which also contains the 3 exonuclease PNPASE  [ **PNP** ]  Recently, RNase E  [ **RNE** ]  has been shown to be able to remove poly A tails by what has been described as an exonucleolytic process that can be blocked by the presence of a phosphate group on the 3 end of the RNA.  Ref: 11328869 Nucleic Acids Res, 2001
**Neutral relationship**- A Streptomyces coelicolor functional orthologue of Escherichia coli RNase E  [ **RNE** ]  shows shuffling of catalytic and PNPase  [ **PNP** ]  binding domains.  Ref: 12675796 Mol Microbiol, 2003
- PNPase  [ **PNP** ]  is not essential, as RNase E  [ **RNE** ]  remains fully active towards mRNAs in  **rne**   **pnp**  mutants.  Ref: 10411735 Mol Microbiol, 1999
- The major RNase E  [ **RNE** ]  cleavage product denoted pSok 6 is rapidly degraded by polynucleotide phosphorylase  [ **PNP** ]  PNPase  [ **PNP** ] .  Ref: 9383156 Mol Microbiol, 1997
- Thus, the RNase E  [ **RNE** ]  cleavage tags pSok 6 for further rapid degradation by PNPase  [ **PNP** ]  from its 3 end.  Ref: 9383156 Mol Microbiol, 1997
- Both PNPase  [ **PNP** ]  and RNase II can degrade the major 3 cleavage product generated by RNase E  [ **RNE** ] .  Ref: 9695924 Microbiology, 1998
- As expected, purified degradosomes, a multi protein complex containing, among others, RNase E  [ **RNE** ] , PNPase  [ **PNP** ] , and RhlB, generate an authentic 147 residue RNase E  [ **RNE** ]  cleavage product from the rpsT mRNA in vitro.  Ref: 9642084 J Mol Biol, 1998
- The ribosomal protein S1, which is known to interact with sequences at the 5 ends of mRNA molecules during the initiation of translation, can bind to both RNase E  [ **RNE** ]  and PNPase  [ **PNP** ] , but in contrast to CspE, did not affect the ribonucleolytic actions of these enzymes.  Ref: 11390393 J Biol Chem, 2001
- The exoribonucleolytic activity of the degradosome was due to polynucleotide phosphorylase  [ **PNP** ] , rather than the recently reported exonucleolytic activity exhibited by a purified fragment of RNase E  [ **RNE** ]  Huang, H.  Ref: 9933592 J Biol Chem, 1999
- Earlier work has shown that the C terminal region of RNase E  [ **RNE** ]  is a scaffold for the binding of degradosome components and has identified specific RNase E  [ **RNE** ]  segments necessary for its interaction with polynucleotide phosphorylase  [ **PNP** ]  PNPase  [ **PNP** ] , RhlB RNA helicase, and enolase.  Ref: 11134527 Proc Natl Acad Sci U S A, 2001
- We also show that the C terminal half of E. coli RNase E  [ **RNE** ]  is both sufficient and necessary for its physical interaction with the 3 5 exoribonuclease polynucleotide phosphorylase  [ **PNP** ] , the RhlB helicase, and the glycolytic enzyme enolase, which are components of a degradosome complex.  Ref: 9751718 Proc Natl Acad Sci U S A, 1998
- Polyribonucleotide phosphorylase PNPase  [ **PNP** ]  is one of the critical components of the E. coli RNA degradosome, which consists of both PNPase  [ **PNP** ]  and endoribonuclease RNase E  [ **RNE** ] .  Ref: 9502433 DNA Cell Biol, 1998
- RNase E  [ **RNE** ]  is a large multidomain protein with N terminal ribonucleolytic activity, an RNA binding domain and a C terminal scaffold that binds PNPase  [ **PNP** ] , enolase and RhlB.  Ref: 12035760 Biochem Soc Trans, 2002
- Our results demonstrate that the carboxy terminal half CTH of ribonuclease E  [ **RNE** ]  RNase E  [ **RNE** ]  contains the binding sites for the three other major degradosomal components, the DEAD box RNA helicase RhlB, enolase, and polynucleotide phosphorylase  [ **PNP** ]  PNPase  [ **PNP** ] .  Ref: 9732274 Genes Dev, 1998
- It was already known that an RNase E  [ **RNE** ]  cleavage occurring at the M2 site, ten nucleotides downstream of the coding sequence of rpsO, removes the 3 hairpin which protects the primary transcript from the attack of polynucleotide phosphorylase  [ **PNP** ]  and RNase II.  Ref: 10047480 J Mol Biol, 1999
- However, transiently increasing intracellular poly A levels has also been shown to stabilize the  **pnp**  and  **rne**  transcripts, leading to increased polynucleotide phosphorylase  [ **PNP** ]  PNPase  [ **PNP** ]  and RNase E  [ **RNE** ]  levels respectively.  Ref: 12207699 Mol Microbiol, 2002

**Non-interactive relationship** (e.g. studied together, co-existance, homology, etc.)

- Strains carrying mutations in the genes encoding RNase E  [ **RNE** ] , polynucleotide phosphorylase  [ **PNP** ]  PNPase  [ **PNP** ] , RNase II and poly A polymerase I PcnB PAP I alone or in combination were used to investigate degradation patterns and relative half lives of CopA.  Ref: 9695924 Microbiology, 1998
- An  **rne**  1  **pnp**  7 double mutation suppresses the temperature sensitive defect of lacZ gene expression in a divE mutant.  Ref: 9515904 J Bacteriol, 1998
- Inactivating mrsC significantly increased the half lives of the trxA, cat, secG, and kan mRNAs, particularly in an mrsC505  **pnp**  7 rnb 500  **rne**  1 multiple mutant.  Ref: 9537393 J Bacteriol, 1998
- These results provide evidence for the first time that both functional and physical interactions of individual degradosome protein components can occur in the absence of RNase E  [ **RNE** ]  and raise the prospect that the RNase E  [ **RNE** ]  independent complexes of RhlB RNA helicase and PNPase  [ **PNP** ] , detected in vivo, may constitute mini machines that assist in the degradation of duplex RNA in structures physically distinct from multicomponent RNA degradosomes.  Ref: 12181321 J Biol Chem, 2002
- The multifunctional ribonuclease RNase E  [ **RNE** ]  and the 3 exonuclease polynucleotide phosphorylase  [ **PNP** ]  PNPase  [ **PNP** ]  are major components of an Escherichia coli ribonucleolytic machine that has been termed the RNA degradosome.  Ref: 11390393 J Biol Chem, 2001
- In addition, experiments using  **pnp**  lacZ and  **rne**  lacZ translational fusions demonstrate that the variations in transcript stability and protein levels arise from alterations in the autoregulation of both genes.  Ref: 12207699 Mol Microbiol, 2002
- DEAD box RhlB RNA helicase physically associates with exoribonuclease PNPase  [ **PNP** ]  to degrade double stranded RNA independent of the degradosome assembling region of RNase E  [ **RNE** ] .  Ref: 12181321 J Biol Chem, 2002
- Here, we have investigated the role that such nucleases play in modulating polyadenylation in vivo by measuring total poly A levels, polyadenylation of specific transcripts, growth rates and cell viabilities in strains containing various amounts of poly A polymerase I PAP I, polynucleotide phosphorylase  [ **PNP** ]  PNPase  [ **PNP** ] , RNase II and RNase E  [ **RNE** ] .  Ref: 10844684 Mol Microbiol, 2000
- Moreover, both the initial RNase E  [ **RNE** ]  dependent cleavage of RNAI and lacZ mRNA and the subsequent attack of RNAI by polynucleotide phosphorylase  [ **PNP** ]  and poly A polymerase were slowed.  Ref: 9600918 Proc Natl Acad Sci U S A, 1998
- Escherichia coli poly A binding proteins that interact with components of degradosomes or impede RNA decay mediated by polynucleotide phosphorylase  [ **PNP** ]  and RNase E  [ **RNE** ] .  Ref: 11390393 J Biol Chem, 2001
- The trans acting proteins are mainly four nucleases, two endo RNase E  [ **RNE** ]  and RNase III and two exonucleases PNPase  [ **PNP** ]  and RNase II, and poly A polymerase.  Ref: 10690408 Annu Rev Genet, 1999
- In Escherichia coli, the exoribonuclease polynucleotide phosphorylase  [ **PNP** ]  PNPase  [ **PNP** ] , the endoribonuclease RNase E  [ **RNE** ] , a DEAD RNA helicase and the glycolytic enzyme enolase are associated with a high molecular weight complex, the degradosome.  Ref: 11680851 RNA, 2001
- Whereas PNPase  [ **PNP** ]  and enolase are present in E. coli in large excess relative to RNase E  [ **RNE** ]  and therefore are detected in cells largely as molecules unlinked to the RNase E  [ **RNE** ]  scaffold, immunogold labeling and biochemical analyses show that helicase is present in approximately equimolar amounts to RNase E  [ **RNE** ]  at all cell growth stages.  Ref: 11134527 Proc Natl Acad Sci U S A, 2001
- We conclude that the C terminal half of RNase E  [ **RNE** ]  is not essential for viability but plays an important role for binding with PNPase  [ **PNP** ] .  Ref: 8682798 J Bacteriol, 1996
- The most important recent developments have been the discovery that the degradosome a multiprotein complex containing an endoribonuclease RNase E  [ **RNE** ] , an exoribonuclease polynucleotide phosphorylase  [ **PNP** ] , and a DEAD box helicase RhlB has a central role in mRNA degradation.  Ref: 10684583 Bioessays, 2000
- Using an E. coli two hybrid system, together with BIAcore apparatus, we investigated the ability of three proteins, polynucleotide phosphorylase  [ **PNP** ]  PNPase  [ **PNP** ] , RhlB RNA helicase, and enolase, a glycolytic protein, to interact physically and functionally independently of RNase E  [ **RNE** ] .  Ref: 12181321 J Biol Chem, 2002
- These results suggest that MrsC, directly or indirectly, controls endonucleolytic processing of mRNAs that may be independent of the RNase E  [ **RNE** ]  PNPase  [ **PNP** ]  RhlB multiprotein complex.  Ref: 9537393 J Bacteriol, 1998
- Furthermore, in the absence of RNase E  [ **RNE** ] , purified RhlB can carry out ATP dependent unwinding of double stranded RNA and consequently modulate degradation of double stranded RNA together with the exonuclease activity of PNPase  [ **PNP** ] .  Ref: 12181321 J Biol Chem, 2002
- Meanwhile, the pools of RNase E  [ **RNE** ]  and polynucleotide phosphorylase  [ **PNP** ] , which, in growing cells, are limited because these RNases autoregulate their own synthesis, cannot expand.  Ref: 9600918 Proc Natl Acad Sci U S A, 1998
- Deletion analysis mapped the catalytic domain of RNase ES near its centre and showed that regions located near the RNase ES termini interact with an S. coelicolor homologue of polynucleotide phosphorylase  [ **PNP** ]  PNPase  [ **PNP** ]  a major component of E. coli RNase E  [ **RNE** ]  based degradosomes.  Ref: 12675796 Mol Microbiol, 2003
- It was also found that introduction of an  **rne**  1  **pnp**  7 double mutation restored the expression of mutant lacZ genes with only UCA codons at position 6 or 462.  Ref: 10913980 Can J Microbiol, 2000
- We found that the defect was almost completely restored by introducing an  **rne**  1  **pnp**  7 double mutation into the divE mutant.  Ref: 9515904 J Bacteriol, 1998
- Polynucleotide phosphorylase  [ **PNP** ] , RNase II and RNase E  [ **RNE** ]  play different roles in the in vivo modulation of polyadenylation in Escherichia coli.  Ref: 10844684 Mol Microbiol, 2000
- Messenger RNA decay in Escherichia coli is slowed in  **pnp**  7 PNPase  [ **PNP** ]  rnb 500 RNase II  **rne**  1 RNase E  [ **RNE** ]  multiple mutants.  Ref: 9159469 J Mol Biol, 1997
- RNase E  [ **RNE** ]  and PNPase  [ **PNP** ]  are found in a multienzyme complex called the degradosome.  Ref: 10690408 Annu Rev Genet, 1999
- The Escherichia coli degradosome is a multienzyme complex with four major protein components the endoribonuclease RNase E  [ **RNE** ] , the exoribonuclease PNPase  [ **PNP** ] , the RNA helicase RhlB and enolase.  Ref: 9383162 Mol Microbiol, 1997
- Our results indicate that RNase E  [ **RNE** ]  associated with the degradosome may contribute to the removal of poly A tails from 5 monophosphorylated RNAs, but this is only likely to be significant should their attack by PNPase  [ **PNP** ]  be blocked.  Ref: 11328869 Nucleic Acids Res, 2001
- Although it was proposed that polyadenylation of RNAs in bacteria leads to their enhanced degradation, in most cases stabilization of these molecules was observed only when other mutations  **pnp** , rnb and  **rne**  were present in the pcnB strain.  Ref: 9710253 FEBS Lett, 1998
- Polyadenylation of Escherichia coli transcripts plays an integral role in regulating intracellular levels of polynucleotide phosphorylase  [ **PNP** ]  and RNase E  [ **RNE** ] .  Ref: 12207699 Mol Microbiol, 2002
- The results are consistent with reports that GroEL can associate with RNase E  [ **RNE** ]  and with other studies showing that RNase E  [ **RNE** ]  and PNPase  [ **PNP** ]  can form complexes.  Ref: 8810258 J Biol Chem, 1996
- Our results demonstrate that a degradosome like complex containing RNase E  [ **RNE** ] , PNPase  [ **PNP** ] , and RhlB can form spontaneously in vitro in the absence of all other cellular components.  Ref: 10521403 Genes Dev, 1999
- This article provides a critical examination of recent progress in the enzymology of mRNA decay in Escherichia coli, focusing on six major enzymes RNase III, RNase E  [ **RNE** ] , polynucleotide phosphorylase  [ **PNP** ] , RNase II, poly A polymerase s, and RNA helicase s.  Ref: 9932452 Prog Nucleic Acid Res Mol Biol, 1999
- Thus, it appears that polyadenylation of transcripts in E. coli serves as a sensing mechanism by which the cell adjusts the levels of both RNase E  [ **RNE** ]  and PNPase  [ **PNP** ] .  Ref: 12207699 Mol Microbiol, 2002
- Here, we show that the half lives of both the  **pnp**  and  **rne**  transcripts are dependent on the intracellular level of polyadenylated transcripts.  Ref: 12207699 Mol Microbiol, 2002
- RNase E  [ **RNE** ]  and PNPase  [ **PNP** ]  of the multiprotein complex presumably cooperate for effective processing and turnover of specific substrates, such as mRNAs and other RNAs in vivo.  Ref: 8682798 J Bacteriol, 1996
- In contrast, the transcripts for  **rne**  RNase E  [ **RNE** ]  and  **pnp**  polynucleotide phosphorylase  [ **PNP** ] , PNPase  [ **PNP** ] , enzymes involved in mRNA decay, were stabilized.  Ref: 10594833 Mol Microbiol, 1999
- The enzymes polynucleotide phosphorylase  [ **PNP** ] , poly A polymerase and RNase E  [ **RNE** ]  have been demonstrated to have roles in both messenger and RNAI decay.  Ref: 10589716 Microbiology, 1999
- To examine whether inactivation of mRNA degradation pathways restores the defect in lacZ gene expression, we constructed divE mutants containing  **rne**  1, rnb 500, and  **pnp**  7 mutations in various combinations.  Ref: 9515904 J Bacteriol, 1998
